# Severe Maternal Hyperglycemia Exacerbates the Development of Insulin Resistance and Fatty Liver in the Offspring on High Fat Diet

**DOI:** 10.1155/2012/254976

**Published:** 2012-04-12

**Authors:** Yong Song, Jibin Li, Yong Zhao, Qijuan Zhang, Zhiguo Liu, Jingna Li, Xiaoyi Chen, Zhu Yang, Chao Yu, Xiaoqiu Xiao

**Affiliations:** ^1^Institute of Life Sciences, Chongqing Medical University, Chongqing 400016, China; ^2^Department of Nutrition and Food Hygiene, School of Public Health and Management, Chongqing Medical University, Chongqing 400016, China

## Abstract

*Background*. Adverse maternal environments may predispose the offspring to metabolic syndrome in adulthoods, but the underlying mechanism has not been fully understood. *Methods*. Maternal hyperglycemia was induced by streptozotocin (STZ) injection while control (CON) rats received citrate buffer. Litters were adjusted to eight pups per dam and then weaned to standard diet. Since 13 weeks old, a subset of offspring from STZ and CON dams were switched to high fat diet (HFD) for another 13 weeks. Glucose and insulin tolerance tests (GTT and ITT) and insulin secretion assay were performed; serum levels of lipids and leptin were measured. Hepatic fat accumulation and islet area were evaluated through haematoxylin and eosin staining. *Results*. STZ offspring exhibited lower survival rate, lower birth weights, and growth inhibition which persisted throughout the study. STZ offspring on HFD showed more severe impairment in GTT and ITT, and more profound hepatic steatosis and more severe hyperlipidemia compared with CON-HFD rats. *Conclusions*. Offspring from diabetic dams would be prone to exhibit low birth weight and postnatal growth inhibition, but could maintain normal glucose tolerance and insulin sensitivity. HFD accelerates development of insulin resistance in the offspring of diabetic dams mainly via a compensatory response of islets.

## 1. Introduction

Metabolic syndrome (MetS) is a clustering of metabolic disorders including hyperglycemia, hypertension, dyslipidemia, overweight, and central obesity, with insulin resistance as the central feature of this syndrome [1]. A global increase in the prevalence of MetS has been found in the last decades due to the worldwide epidemic of obesity [2, 3]. The risk of the MetS depends on genetic susceptibility but is modulated by pre- and postnatal environmental factors. A growing body of evidence suggests that adverse environmental conditions during crucial periods of development may predispose individuals to develop different components of the MetS in adulthood [4]. Of particular note is maternal diabetes, including type 1 and type 2 diabetes and gestational diabetes mellitus (GDM). An overall increasing prevalence of diabetes indicates an emergence of diabetes among childbearing women. During pregnancy, especially during critical window periods for organogenesis and fetal development, unsatisfactory glycemic control is known to increase the incidence of severe obstetrical complications, including preeclampsia, macrosomia, and Caesarean section. Additionally, intrauterine exposure to a hyperglycemic environment predisposes the offspring to develop glucose intolerance and obesity in early life [5–9]. These epidemiological and clinical studies have strongly certified that maternal pregnancy with hyperglycemia was a major risk factor for the development of MetS in their offspring [10, 11]. Thus, it seems that the key point is to evaluate how maternal hyperglycemia during pregnancy affects fetal growth and organ development, and whether improved glycemic control may ameliorate pregnancy outcome.

Animal models provide good opportunities to observe the long-term outcomes of maternal hyperglycemia during periods of pregnancy and lactation in the offspring and to further elucidate the underlying mechanism. A variety of such murine models have been established using pregnant animals by administration of streptozotocin, an antibiotic agent with specific toxicity to pancreatic *β*-cells [12–21]. However, the majority of these animal models were employed to evaluate mild diabetic effects, and mostly in middle or later stages of pregnancy with hyperglycemia. In humans, severe hyperglycemia has been reported in some pregnant women with uncontrolled diabetes, where microsomia is most often observed [22]. In this study, we investigated maternal-fetal outcomes of the severe maternal hyperglycemia (>20 mM) during the whole periods of pregnancy and lactation and assessed the metabolic features in their offspring.

## 2. Materials and Methods

### 2.1. Animals and Treatment

Sprague-Dawley rats (8-week-old, weighing 200–250 g) were used in this study. All rats were housed individually in wood-chip-bedded plastic cages at constant temperature (22 ± 1°C) and humidity (60 ± 5%) with a 12-hour light-dark cycle. They had free access to water and a standard chow diet (10% of energy from fat, SLAC laboratory animal, China). After mating, the first day of gestation (G0) was defined by the presence of spermatozoids in vaginal smears. Pregnant rats were made diabetic by a single intraperitoneal injection of streptozotocin (Sigma, 40 mg/kg body weight) dissolved in 0.1 M citrate buffer, pH 4.3, on G0, and control rats were injected with the same volume of citrate buffer. Maternal blood glucose levels were monitored by glucose meter (One Touch, Johnson, USA). Blood samples were obtained by cutting off the tip of tails and squeezing gently on days of G5, G12, and G19, as well as on day 10 of lactation (L10). Only those rats with blood glucose levels above 20 mM on all four time points were designated as severe maternal hyperglycemia. A total of 10 hyperglycemic and 6 control dams were included in the study. All rats were allowed to deliver spontaneously and litter size was examined, and the pups were weighed within 12 hours after birth and then returned to their mothers immediately. On the first day, litters were adjusted to 8 pups per dam. To exclude the influence of female hormones, only male offsprings were used in the present study. Offsprings were fed the same standard chow diet at 3 weeks of age after weaning and body weights were recorded biweekly. From 13 weeks of age, half of the offsprings in both groups were switched to high fat diet (HFD, 45% of energy from fat, SLAC laboratory animal, China), and the rest were maintained on standard chow diet, and thereby four separate adult offspring groups were studied: CON offspring fed standard chow diet (CON-S), CON offspring fed HFD (CON-H), STZ offspring fed standard chow diet (STZ-S), and STZ offspring fed HFD (STZ-H). All offspring were sacrificed at 25 weeks of age, and plasma was immediately isolated from blood samples by centrifugation at 3000 rpm for 20 minutes at 4°C and stored at −80°C for analysis. Epididymal white adipose tissue, liver, and pancreas were quickly removed and dissected free of connective tissues and used for RNA and protein extraction, or for histological study. All experimental procedures were approved by the Institutional Animal Care and Use Committee of Chongqing Medical University.

### 2.2. Glucose Tolerance Tests, Insulin Tolerance Tests, and Insulin Releasing Tests

Glucose tolerance tests (GTTs) were performed at 8 and 24 weeks of age, and insulin tolerance tests (ITTs) and insulin releasing tests (IRTs) were performed at 24 weeks. After 16 hours fasting, GTT was performed as follows: after collecting blood from the tail (0 time), the rats received glucose (2 g/kg body weight) via intraperitoneal injection and blood glucose was measured at 0, 15, 30, 60, 120 minutes points as previously reported. IRT was determined during the GTT, at 0, 30, 60, 90 minutes, and an aliquot of the blood sample (>100 *μ*L) was collected for serum by centrifugation. Serum insulin was determined with a highly specific enzyme-linked immunosorbent assay (ELISA) kits as per the manufacturer's directions (R&D, USA). ITT was performed after 4 hours fasting, human recombinant insulin (1units/kg, Novo Nordisk, Denmark,) was intraperitoneally injected and blood glucose was measured at 0, 30, 60, 90, 120 minutes.

### 2.3. Serum Assays

Serum leptin was determined with a rat leptin ELISA immunosorbent assay kit (R&D, USA). Serum total cholesterol (TC), triglyceride (TG), high-density lipoprotein (HDL), and low-density lipoprotein (LDL) were measured with Biochemical Analyzer (SIEMENS ADVIA 2400 Chemistry System, Germany) [23].

### 2.4. Tissue Collection and Histological Studies

Liver, pancreas, gonadal and retroperitoneal white adipose tissue were removed, washed with cold saline (0.9%), and weighed immediately. The liver and pancreas tissues were fixed in 10% formalin for 24 hours, ethyl alcohol dehydrated, and embedded in paraffin. Tissues were cut to 6 fixed sections (5 *μ*m), stained with hematoxylin and eosin (H&E). Three fixed pancreas sections per animal and three different rats in each group were studied. Sections were photoed by microscope at a magnification of 200x (Olympus BX51, Japan) and pancreatic islets area was quantified using Image J 1.40 software.

### 2.5. Statistical Analysis

All data were analyzed by using SAS 9.1 software (SAS, USA). Survival rate was compared by rank sum test. Two group's data were expressed as mean ± SEM and compared by paired sample *t*-test analysis of variance (ANOVA). Two-way ANOVA was used to assess interaction between maternal glycemic type and offspring fed-type. A *P *value <0.05 was considered statistically significant.

## 3. Results

### 3.1. A Single Dose of STZ Induces Persistent Hyperglycemia

**Figure 1:**
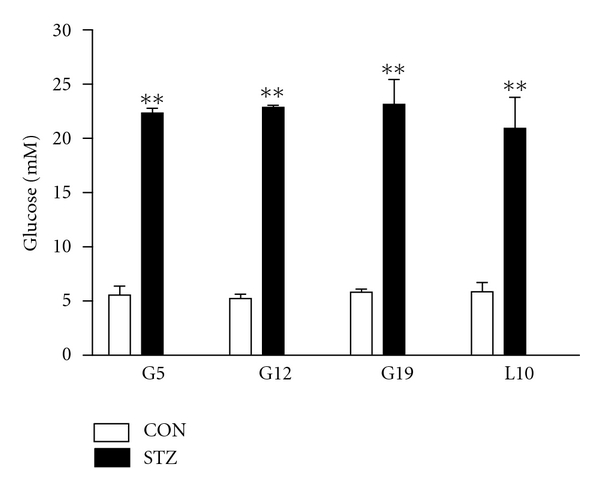
Maternal blood glucose levels in citrate buffer-treated control dams (CON, *n* = 6) or streptozotocin-treated dams (STZ, *n* = 10) on days 5, 12, and 19 of the gestation (G5, G12, and G19) and day 10 of lactation (L10). Data are expressed as means ± SEM; **P < 0.01 versus CON.

Among the 15 pregnant rats injected with STZ, only 10 rats (66.7%) met our criteria with blood glucose levels all above 20 mM throughout gestational and lactation periods, and glucose levels were 23.3 ± 0.4 mM, 22.8 ± 0.2 mM, 23.1 ± 2.3 mM, and 20.9 ± 2.4 mM on G5, G12, G19, and L10, respectively. However, citrate buffer-treated CON dams displayed normal blood glucose levels, which were 5.6 ± 0.8 mM, 5.2 ± 0.4 mM, 5.8 ± 0.3 mM, and 5.9 ± 0.8 mM, on G5, G12, G19, and L10, respectively (Figure 1).

### 3.2. Severe Maternal Hyperglycemia during Gestation Alters Perinatal Outcomes

**Figure 2:**
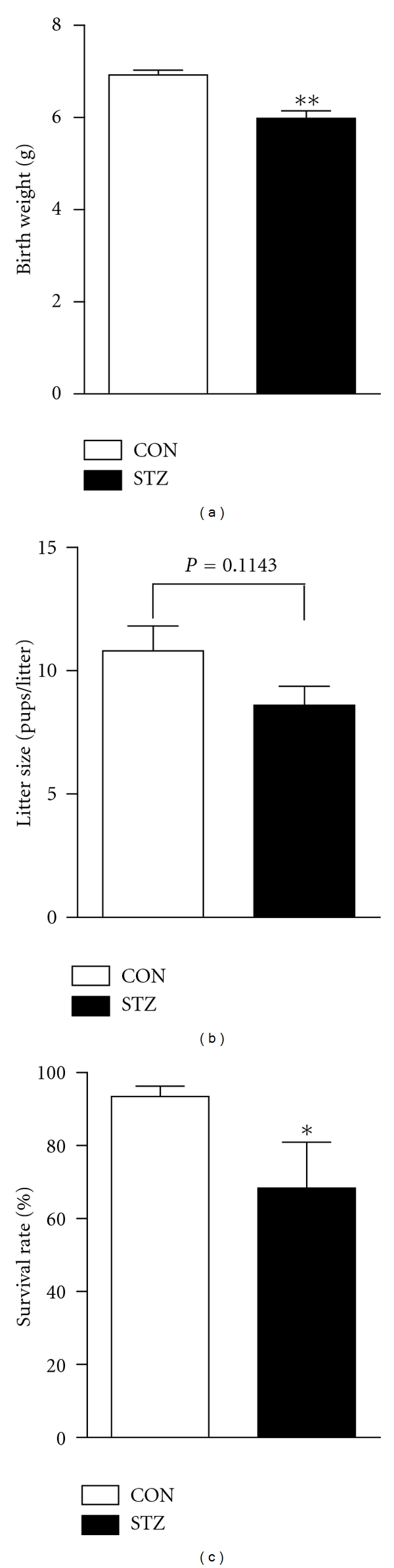
Maternal STZ treatment during gestation alters perinatal outcomes. Birth weight (a), litter size (b), and survival rate of pups at 7-day-age (c) were monitored for the offspring from citrate buffer-treated control dams (CON, *n* = 54) or streptozotocin-treated dams (STZ, *n* = 71). Values are means ± SEM; statistically significant differences are indicated by *P < 0.05 and **P < 0.01 compared with CON.

Compared with CON offspring, birth weight of STZ offspring tended to be reduced (STZ offspring, 6.0 ± 0.2 g; CON offspring 6.9 ± 0.1 g; *P* < 0.01, Figure 2(a)). However, severe maternal hyperglycemia induced by STZ administration did not significantly alter the litter size (STZ, 8.6 ± 0.8; CON, 10.8 ± 1.0, *P* = 0.1143, Figure 2(b)). Furthermore, the survival rate from STZ offsprings within the first week of postpartum was significantly lower than that of CON offspring (*X* ^2^ = 6.3, *P* < 0.05, Figure 2(c)). Other perinatal outcomes did not differ between the two groups.

### 3.3. Severe Maternal Hyperglycemia during Gestation and Lactation Impairs Postnatal Growth Rate in the Offspring

**Figure 3:**
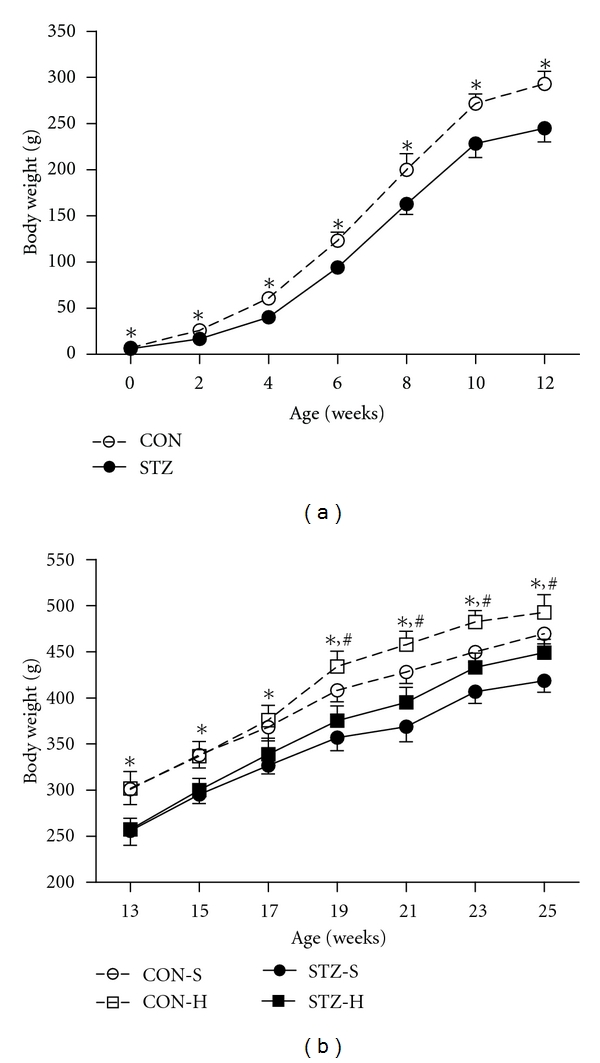
Severe maternal hyperglycemia impairs postnatal growth rate in the offspring. (a) Bodyweight gain in CON offspring and STZ offspring from birth to 12 weeks under standard chow diet feeding after weaning, data were mean ± SEM, *P < 0.05 versus STZ; (b) bodyweight gain in CON offspring and STZ offspring under standard chow diet or high fat diet feeding from 13 weeks to 25 weeks. CON-S: CON offspring fed with standard chow diet; CON-H: CON offspring fed with high fat diet; STZ-S: STZ offspring fed with standard chow diet; STZ-H: STZ offspring fed with high fat diet. Data were presented as means ± SEM. *P < 0.05 versus CON-S, ^#^ *P* < 0.05 versus STZ-H. *n* = 6–11 in each group.

The STZ offspring had significantly lower body weight than CON offspring at the first 12 weeks of age when fed standard chow diet since weaning (Figure 3(a)). The body weight difference between two groups still existed when fed HFD for another 13 weeks, and CON-H offspring were 9.6% heavier than STZ-H offspring (Figure 3(b)). These data indicated that severe maternal hyperglycemia during the periods of gestation and lactation impaired postnatal growth in their offspring.

### 3.4. Severe Maternal Hyperglycemia during Gestation and Lactation Alters Glucose Disposal and Insulin Sensitivity in the Offspring

**Figure 4:**
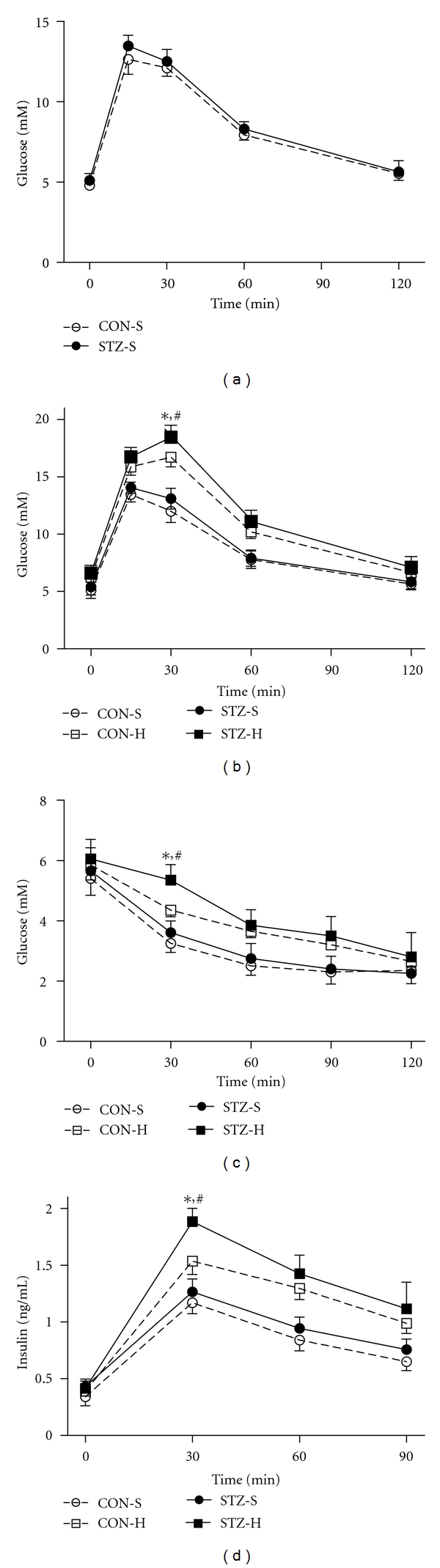
Severe maternal hyperglycemia during gestation and lactation produces metabolic dysfunction only in STZ offspring on HFD. (a) Glucose tolerance test (GTT) at 8 weeks between CON offspring and STZ offspring under standard chow diet feeding. (b) GTT at 24 weeks in CON offspring and STZ offspring under standard chow diet or high fat diet feeding. (c) Insulin tolerance test at 25 weeks in these four groups. (d) Insulin releasing test determined during the GTT. CON-S: CON offspring fed with standard chow diet; CON-H: CON offspring fed with high fat diet; STZ-S: STZ offspring fed with standard chow diet; STZ-H: STZ offspring fed with high fat diet. Data were presented as means ± SEM. *P < 0.05 versus STZ-S, ^#^ *P* < 0.05 versus CON-H, *n* = 6–11 in each group.

At the age of 8 weeks, both STZ and CON offsprings fed with chow diet exhibited similar pattern as to GTT (Figure 4(a)), which persisted to the age of 24 weeks (Figure 4(b)). However, when maintained on HFD, STZ-H rats displayed significantly higher glucose levels at 30 min during GTT when compared with CON-H offspring (Figure 4(b)). Meanwhile, serum insulin levels at 30 min during GTT were significantly higher in STZ-H offspring (Figure 4(c)). To further examine insulin sensitivity in these rats, we performed insulin tolerance test. Thirty minutes after insulin injection, blood glucose levels of STZ-H animals were significantly higher than those of other counterparts (Figure 4(d)). These data suggested that severe maternal hyperglycemia during periods of gestation and lactation produced metabolic dysfunction in their offspring only on high fat diet but not on normal diet.

### 3.5. Severe Maternal Hyperglycemia during Gestation and Lactation Alters Islet Morphology in the Offspring

**Figure 5:**
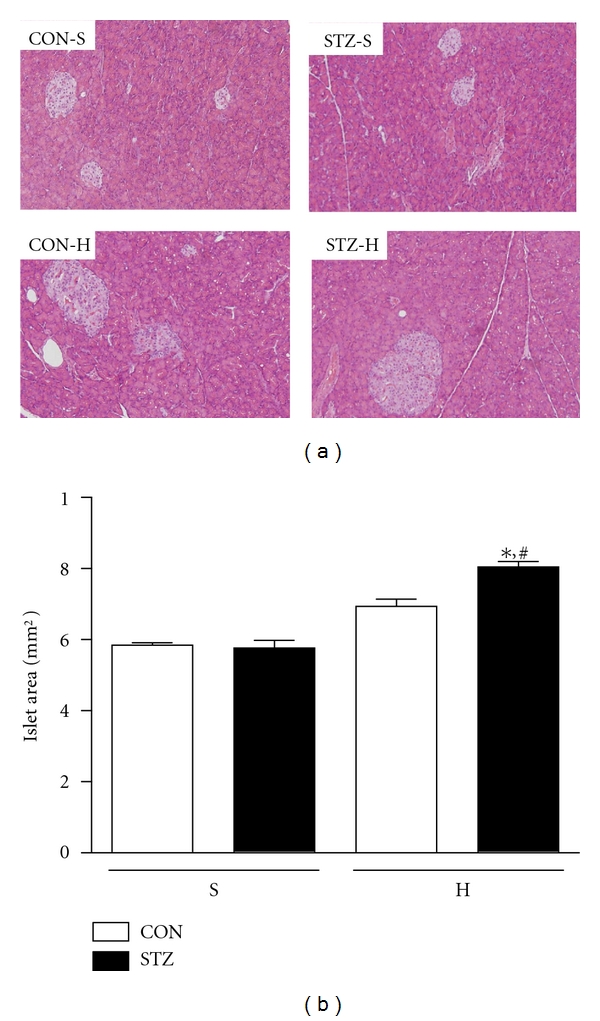
Severe maternal hyperglycemia during gestation and lactation alters islet morphology and lipid metabolism in the offspring. (a) Histological sections of hematoxylin and eosin (H&E)-stained pancreas of CON offspring and STZ offspring under standard chow (S) or high fat diet (H) feeding. Sections were examined by light microscopy at a magnification of 200x; (b) islets areas were quantified using Image J 1.40 software. Values were means ± SEM; *P < 0.05 versus STZ-S, ^#^ *P* < 0.05 versus CON-H; *n* = 5 in each group.

We then examined islet area to explore the mechanisms of impaired insulin sensitivity and secretion. As shown in Figure 5(a), the islet size did not differ between STZ and CON offspring on standard chow diet. HFD feeding increased the islet sizes in both CON and STZ offspring. Interestingly, islet sizes of STZ-H were significantly larger than those of CON-H (Figures 5(a) and 5(b)). These results suggested that high fat diet might induce a compensatory response to maintain normal islet function in STZ offspring.

### 3.6. Severe Maternal Hyperglycemia during Gestation and Lactation Alters Lipid Metabolism in the Offspring

**Figure 6:**
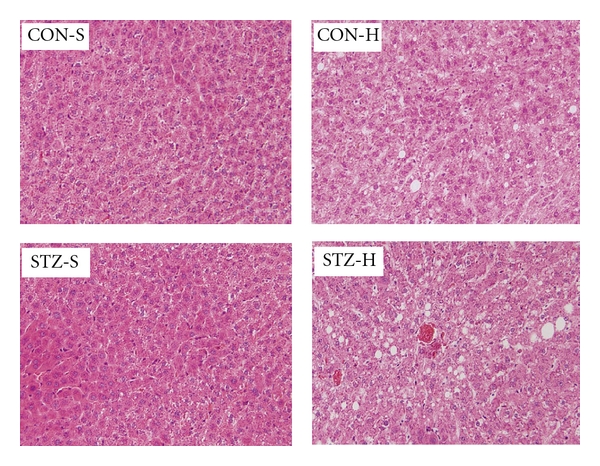
Severe maternal hyperglycemia during gestation and lactation alters liver fat accumulation in the offspring. Histological sections of hematoxylin and eosin (H&E)-stained liver of CON offspring and STZ offspring under standard chow diet feeding. Sections were examined by light microscopy at a magnification of 200x; CON-S: CON offspring fed with standard chow diet; CON-H: CON offspring fed with high fat diet; STZ-S, STZ offspring fed with standard chow diet; STZ-H: STZ offspring fed with high fat diet. *n* = 5 in each group.

General biochemical characteristics of the animals at the age of 25 weeks were summarized in Table 1. Even HFD feeding increased the sum of WAT in both CON and STZ offspring, STZ offspring had lower WAT than CON offspring. As expected, serum leptin level of the two groups tended to be the similar trends. Compared with other three groups, STZ-H animals had significantly higher levels of TG and LDL, while significantly lower HDL level (Table 1). In STZ-H animals, there were more manifest hepatic steatosis, which was correspondent with the obvious dyslipidemia in this group (Table 1 and Figure 6). Taken together, these results suggested severe maternal hyperglycemia during pregnancy and lactation induced hepatic steatosis and hyperlipidemia only in their offspring fed with HFD.

## 4. Discussion

Fetal and early postnatal exposure to excess nutritional environment is a well-defined risk factor for the development of MetS [11, 24, 25]. Unlike the majority of previous studies which mainly evaluated the effects of mild hyperglycemia during the middle and later stages of pregnancy, we investigated maternal-fetal outcomes of the severe hyperglycemia (>20 mM) during the whole periods of pregnancy and lactation. As expected, severe diabetic pregnancies lead to disadvantage perinatal outcomes, as reflected by increased perinatal mortality. In addition, our model results in a lower birth weight and impaired postnatal growth in survived offspring of diabetic mothers, and the change in weight gain persisted after weaning and throughout their entire life. When maintained on standard chow diet, offsprings from diabetic mothers exhibited relatively normal glucose tolerance as their counterparts from normal glycemic dams. However, when these rats were fed with HFD, they displayed significantly impaired glucose tolerance, severe insulin insensitivity and fatty liver. This is, to our knowledge, the first report that offspring from diabetic mothers respond differently when challenged with high caloric diets during adulthood and are susceptible to HFD-induced metabolic perturbation.

Although numerous experimental studies have been performed as insulin resistance in offspring from diabetic maternal rats after administration of STZ, results from this model are inconsistent and divergent [12, 15, 16, 19, 20, 26–30]. Discord phenotypes in the offspring born to diabetic dams may possibly be attributable to the doses of STZ, the severity of hyperglycemia, and time windows and duration occurring during pregnancy. Plagemann et al. used a rat model with moderate impairment in glucose tolerance in pregnant rats by application of low dose of STZ on the day of conception and found that offspring of these mildly diabetic mothers developed early overweight and hyperinsulinemia accompanied by early signs of insulin resistance [27]. With the similar protocol, the findings were further confirmed by several other studies [16, 29]. López-Soldado and Herrera showed that rats displayed a different response to STZ when administered at the onset of pregnancy. At the dose of 35 mg/kg, STZ induced both mild (glucose levels around 11 mM) and severe (22 mM) hyperglycemia, and the phenotypes of offspring born to these moderately or severely hyperglycemic dams were quite different. Offspring from moderately hyperglycemic dams were macrosomic, while pups from severely hyperglycemic mothers were smaller, weighed less, and had slightly impaired glucose tolerance [16]. Timing is another important factor. STZ administration before pregnancy may affect fertility and impair embryo development during the preimplantation period [31]. However, if STZ was injected after pregnancy, the chemical may cross the placenta and directly affect the fetus. Therefore, we choose to inject STZ at the onset of pregnancy and select those rats with severe hyperglycemia to mimic the human pregnant women with uncontrolled diabetes. In current study, we observed that offspring born to and nurtured by mothers with severe diabetes exhibited a lower birth weight and impaired postnatal growth, and this change in weight gain persisted after weaning and throughout the entire study, agreeing with previous reports [15, 16]. To determine if animals born to mothers with severe diabetes have similar response with their counterparts from normal glycemic dams after high energy challenge, we fed rats with HFD. As expected, offspring from both STZ and CON mothers showed the same extent of weight gain, although the final body weight was still lower than that of CON offspring after 13 weeks of HFD. Importantly, compared with CON offspring, STZ offspring displayed more severe impairment in glucose tolerance test, and greater insulin insensitivity, dyslipidemia, and hepatosteatosis on HFD, while the metabolic dysfunctions were not obvious in STZ offspring on standard chow diets [32]. All these findings indicated that severe maternal hyperglycemia during periods of both gestation and lactation might contribute to the increased risk for developing MetS in later life of their offspring.

The mechanisms underlying how maternal hyperglycemia during early life stage affects organ development, fetal and postnatal growth and metabolic homeostasis in offspring remain unclear. However, an adaptive compensatory response of pancreatic islets may provide a tentative explanation for their features in glucose homeostasis. It is generally accepted that when fetus exposed to hyperglycemic environment, a large number of maternal glucose would cross through the placental barrier into the fetus, to stimulate the proliferation of fetal islet cells, and thereby insulin secretion was increased and fetal hyperglycemia and hyperinsulinemia were produced. On the other hand, maternal hyperglycemia can reduce the oxygen supply from placenta to the fetus, and fetal hyperglycemia may induce increase in fetal oxygen consumption. The chronic hypoxia leads to intrauterine fetal growth restriction. Rodríguez reported that immediately after parturition, pancreatic islets in pups from mildly and severely diabetic mothers were smaller than those in neonates from nondiabetic controls but the size was rapidly normalized when exposure to high blood sugar had disrupted [33]. The similar results were also observed in the Goto-Kakisaki (GK) rat, a well-defined genetic model for type 2 diabetes [34]. When the offspring from diabetic mothers were fed relatively-healthy diets, these animals can maintain normal glucose levels. The phenomenon was also observed in our current studies and was in agreement with some clinical observations [35, 36]. Moreover, our data presented additional evidence that when offspring born to and nurtured by severe diabetic mothers were fed with high-fat-diet, a mild, but significant increase in islets area was found, which presumably was via a compensatory response of *β*-cell mass [37]. Therefore, we speculated that exposure *in utero* to a severe hyperglycemia environment exacerbates the development of insulin resistance in offspring with high fat feeding.

In summary, we observed that severe maternal diabetes during gestation and lactation lead to microsomic offspring and postnatal growth inhibition. These animals can maintain normoglycemia and relatively normal insulin sensitivity under standard diet. HFD accelerates the development of insulin resistance, dyslipidemia, and hepatosteatosis in the offspring of diabetic dams. Thus, these results reinforce the importance for women to avoid exposure to hyperglycemia before they become pregnant. This rat model with moderate amount of STZ could also be used to study other pathophysiological consequences of the maternal diabetic conditions on the next generation.

## Figures and Tables

**Table 1:** Metabolic parameters of CON offspring and STZ offspring at the age of 25 weeks.

	CON-S	CON-H	STZ-S	STZ-H
Body weight (g)	469.5 ± 20.9	492.7 ± 19.3	418.7 ± 12.6	449.1 ± 19.4^∗,#^
Sum of WAT(g)	20.9 ± 1.9	27.5 ± 0.8	13.3 ± 0.7	17.6 ± 1.2^∗,#^
Insulin (ng/ml)	0.23 ± 0.04	0.39 ± 0.04	0.25 ± 0.05	0.42 ± 0.05*
Leptin (ng/ml)	1.37 ± 0.12	1.95 ± 0.13	1.31 ± 0.21	1.53 ± 0.40
TC (mM)	1.63 ± 0.05	2.27 ± 0.35	1.78 ± 0.37	2.41 ± 0.11*
TG (mM)	5.32 ± 1.02	4.85 ± 1.07	4.50 ± 2.49	8.15 ± 1.64^∗,#^
HDL (mM)	0.80 ± 0.10	1.11 ± 0.11	0.77 ± 0.06	0.59 ± 0.08^∗,#^
LDL (mM)	0.31 ± 0.02	047 ± 0.08	0.37 ± 0.14	0.64 ± 0.06^∗,#^

Sum of WAT, sum of retroperitoneal and gonadal white adipose tissue; TC: total cholesterol; TG: triglyceride; HDL: high-density lipoprotein; LDL: low-density lipoprotein. CON-S: CON offspring fed with standard chow diet; CON-H: CON offspring fed with high fat diet; STZ-S: STZ offspring fed with standard chow diet; STZ-H: STZ offspring fed with high fat diet. Data were presented as means ± SEM. *P < 0.05 versus STZ-S, ^#^ *P* < 0.05 versus CON-H, *n* = 6–11 in each group.
